# Focus on *RAS* Codon 61 Mutations in Metastatic Colorectal Cancer: A Retrospective Analysis

**DOI:** 10.3390/cancers16050988

**Published:** 2024-02-29

**Authors:** Francesco Schietroma, Annunziato Anghelone, Giustina Valente, Viria Beccia, Giulia Caira, Alexia Spring, Giovanni Trovato, Armando Di Bello, Anna Ceccarelli, Laura Chiofalo, Serena Perazzo, Maria Bensi, Angelo Minucci, Andrea Urbani, Luigi Maria Larocca, Michele Basso, Carmelo Pozzo, Lisa Salvatore, Maria Alessandra Calegari, Giampaolo Tortora

**Affiliations:** 1 Medical Oncology, Università Cattolica del Sacro Cuore, 00168 Roma, Italy; francesco.schietroma01@icatt.it (F.S.); annunziato.anghelone@ospedalerc.it (A.A.); giustina.valente01@icatt.it (G.V.); viria.beccia01@icatt.it (V.B.); giulia.caira01@icatt.it (G.C.); alexia.spring01@icatt.it (A.S.); giovanni.trovato01@icatt.it (G.T.); armando.dibello01@icatt.it (A.D.B.); anna.ceccarelli01@icatt.it (A.C.); laura.chiofalo01@icatt.it (L.C.); serena.perazzo01@icatt.it (S.P.); maria.bensi01@icatt.it (M.B.); lisa.salvatore@policlinicogemelli.it (L.S.); giampaolo.tortora@policlinicogemelli.it (G.T.); 2 Departmental Unit of Molecular and Genomic Diagnostics, Genomics Core Facility, Gemelli Science and Technology Park (G-STeP), Fondazione Policlinico Universitario Agostino Gemelli IRCCS, 00168 Roma, Italy; angelo.minucci@policlinicogemelli.it; 3 Clinical Chemistry, Biochemistry and Molecular Biology Operations, Fondazione Policlinico Universitario Agostino Gemelli IRCCS, 00168 Roma, Italy; andrea.urbani@policlinicogemelli.it; 4 Patologia Oncoematologica, Dipartimento di Scienze della Salute della Donna, del Bambino e di Sanità Pubblica, Fondazione Policlinico Universitario Agostino Gemelli IRCCS, 00168 Roma, Italy; luigimaria.larocca@policlinicogemelli.it; 5 Medical Oncology, Comprehensive Cancer Center, Fondazione Policlinico Universitario Agostino Gemelli IRCCS, 00168 Roma, Italy; michele.basso@policlinicogemelli.it (M.B.); carmelo.pozzo@policlinicogemelli.it (C.P.)

**Keywords:** colorectal adenocarcinoma, RAS, metastatic colorectal cancer, RAS signaling, Codon 61, MAPK pathway, RAS effectors, KRAS inhibitors, resistance

## Abstract

**Simple Summary:**

Codon 61 *RAS* mutations are rare in metastatic colorectal cancer. Despite being associated with primary and acquired resistance to anti-EGFR agents, little is known about their phenotype and prognostic impact. We retrospectively investigated the clinicopathological features and prognoses of 50 patients with tumors harboring codon 61 *RAS* mutations compared to 648 codon 61 *RAS* wild-type tumors. We identified a significant correlation between codon 61 *RAS* mutations and metastatic involvement of the peritoneum and ovary and a negative prognostic impact. This is the first evidence of an impact of *RAS* mutational status on the metastatization pattern. These results are of great interest given the high frequency of codon 61 *RAS* mutations as mechanisms of secondary resistance to anti-EGFRs and the advent of RAS inhibitors. This is the widest codon 61 *RAS*-mutated cohort reported so far; nevertheless, these findings must be validated in larger studies.

**Abstract:**

*RAS* mutations involving codon 61 are rare in metastatic colorectal cancer (mCRC), accounting for only 1–4%, but they have recently been identified with high frequency in the circulating tumor DNA (ctDNA) of patients with secondary resistance to anti-EGFRs. This retrospective monocentric study aimed to investigate the clinical phenotype and prognostic performance of codon 61 *RAS*-mutated mCRC. Fifty patients with codon 61 *RAS*-mutated mCRC treated at our institution between January 2013 and December 2021 were enrolled. Additional datasets of codon 61 *RAS* wild-type mCRCs (648 patients) were used as comparators. The endpoint for prognostic assessment was overall survival (OS). Metastatic involvement of the peritoneum or ovary was significantly more frequent in codon 61 *RAS*-mutated mCRC compared to codon 61 *RAS* wild-type (54 vs. 28.5%), non-codon 61 *RAS*-mutated (35.6%), *BRAF* V600E-mutated (25%), and *RAS*/*BRAF* wild-type (20.5%) cohorts. At a median follow up of 96.2 months, the median OS for codon 61 *RAS*-mutated patients was significantly shorter compared to *RAS*/*BRAF* wild-type (26.9 vs. 36.0 months, HR 0.56) patients, while no significant difference was observed compared to non-codon 61 *RAS*-mutated and *BRAF* V600E-mutated patients. We showed a negative prognostic impact and a statistically significant correlation between codon 61 *RAS* mutations and metastatic involvement of the peritoneum and ovary.

## 1. Introduction

Nowadays, while the molecular classification of colorectal cancer (CRC) is becoming more and more complex [1], rat sarcoma virus (*RAS*) mutational status remains a key determinant in every turning point in patients’ therapeutic algorithm [2]. Together with *NRAS* and *HRAS*, *KRAS* is a gene belonging to the *RAS* family, which encodes guanosine-5′-triphosphate (GTP)-binding proteins, important effectors of ligand-bound epidermal growth factor receptor (EGFR) signaling through the mitogen-activated protein kinase (MAPK) axis [3]. *KRAS* mutations affect approximately 30–40% of metastatic CRC (mCRC), with mutations involving codons 12 and 13 being the most represented, occurring in about 85–90% of cases [3,4]. Several studies have demonstrated their role as predictive biomarkers of resistance to anti-EGFR agents [5,6,7]. Hence, all patients diagnosed with mCRC require *RAS* profiling before the administration of anti-EGFRs agents (cetuximab or panitumumab) [8,9,10,11,12,13,14].

Codon 61 mutations are less prevalent, affecting 1–4% of patients with mCRC. Similarly to other *RAS* mutations, these alterations are responsible for a constitutive activation of the RAS/RAF/MAPKs pathway, resulting in oncogenic activity and cell proliferation [15]. Furthermore, in *KRAS* codon 12 and 13, wild-type mCRCs codon 61 mutations have been linked to resistance to anti-EGFR therapies [16,17]. Recently, codon 61 variants have been identified with high frequency in the circulating tumor DNA (ctDNA) of patients with mCRC with secondary resistance to anti-EGFR agents [18,19,20,21], with a prevalence of 50% in the CHRONOS trial [18]. Other rare *KRAS* mutations involve exon 4, codon 117, and codon 146. Similarly to more frequent *RAS* mutations, mutations involving codon 117 and 146 have been associated with resistance to anti-EGFRs therapies [22,23]. Moreover, a large analysis showed a higher incidence of codon 117 and 146 in older patients [24].

Despite its growing clinical relevance, little is known about the clinicopathological and molecular features and prognosis of mCRCs harboring *RAS* codon 61 mutations and their differences with more common codon 12 and 13 mutations, as well as and their impact on prognosis. In 2014, a cohort study by Imamura et al. [25] reported the clinicopathological and molecular features of 19 *KRAS* codon 61-mutated mCRC to be similar to *KRAS* codon 12- and 13-mutated mCRCs. Another study found a weak tendency for peritoneum localization in a population of 14 patients with codon 61 *RAS-*mutated CRC [26]. In our study, we aimed to further investigate the clinical characteristics and prognosis of patients with mCRC harboring *RAS* codon 61 mutations treated at our institution compared to those harboring other non-codon 61 *RAS*-mutated and wild-type tumors.

## 2. Materials and Methods

This is an observational, retrospective, monocentric study. The study was approved by the local Ethics Committee of Fondazione Policlinico Universitario Agostino Gemelli IRCCS, Rome, Italy (protocol number 0054049/2019 18 December 2019). The objective of the study was to investigate and describe clinical phenotype and prognostic performance of mCRCs harboring *RAS* codon 61 mutations.

We examined the medical records of patients diagnosed with mCRC who were treated at our center from January 2013 through December 2021. Eligible subjects were those patients whose tumors carried mutations involving codon 61 of *RAS* gene and were evaluable for survival after at least one line of therapy. We collected data regarding bbaseline demographic and clinical characteristics, first-line treatment, and survival from medical records, while histological reports were used to gather pathological and molecular data. The following baseline demographic and clinical characteristics were collected: sex, age, Eastern Cooperative Oncology Group performance status (ECOG PS) at diagnosis, primary tumor location, onset of metastatic disease, number of metastatic sites, site of metastases, presence of peritoneal and/or adnexal metastases, mucinous histology, grade of differentiation, *RAS*/*BRAF* mutational status, microsatellite instability/mismatch repair (MSI/MMR) status, treatments received (surgery, neoadjuvant or adjuvant chemotherapy, first-line chemotherapy), investigator-assessed best response according to Response Evaluation Criteria in Solid Tumors (RECIST) 1.1 criteria, and survival. *RAS* and *BRAF* mutational status was assessed by means of next-generation sequencing (NGS) or pyrosequencing on formalin-fixed, paraffin-embedded (FFPE) archival tumor tissue samples from primary tumor or metastases. Expression of MMR proteins (MLH1, MSH2, MSH6, and PMS2) was performed via immunohistochemistry. MSI status was assessed via NGS.

Additional datasets of patients affected by mCRC without codon 61 *RAS* mutations (codon 61 *RAS* wild-type) treated at out center during the same time frame were used as comparators. Among this group of patients, we identified three different molecular subgroups, which included, respectively, patients with an *RAS*-mutated disease not involving codon 61 (non-codon 61 *RAS*-mutated mCRCs), patients with mCRC harboring a *BRAF* V600E mutation (*BRAF* V600E-mutated mCRCs), and patients with an *RAS* and *BRAF* wild-type disease (*RAS*/*BRAF* wild-type mCRCs).

For categorial data, counts and percentages were reported using a descriptive method; for continuous variables, median and range were provided. Fisher’s exact test or the chi-square test, when applicable, were used to compare group differences for categorical variables. Overall survival (OS), defined as the time occurring between the diagnosis of metastatic disease to the date of death from any cause, was the endpoint for prognostic analysis. All patients were followed up until death or the time of database lock (January 2023). Patients not experiencing events were censored at the date of last follow up. Survivals were estimated with the Kaplan–Meier method and compared using the log-rank test. Statistical significance was set at *p* = 0.05. Statistical analyses were performed using MedCalc version 14.8.1.

## 3. Results

Between January 2013 and January 2023, a total of 50 patients with a diagnosis of mCRC harboring an *RAS* codon 61 mutation were included in our analysis. Of those, 28 mutations (56%) affected *KRAS* and 22 (44%) *NRAS*. Patients and disease characteristics are summarized in Table 1.

Median age at diagnosis was 65 years (range 34–86 years). Nineteen patients were males (38%) and thirty-one were females (62%). Patients were mainly in good clinical conditions at the time of diagnosis (88% with an ECOG PS 0 or 1). Thirty-six patients (72%) had a left-sided primary tumor, and thirty-three patients (66%) had a synchronous metastatic disease. The most frequent site of metastases was liver (24 patients, 48%), followed by peritoneum or ovary (16 patients, 32%), lymph nodes (15 patients, 30%), and lungs (11 patients, 22%). Moreover, 27 patients (54%) developed metastases involving the peritoneum or ovary during their clinical history. The majority of patients received resection of primary tumor (40 patients, 80%). Twenty nine patients (58%) underwent a first-line therapy which included bevacizumab. As chemotherapy regimen, twenty nine patients (58%) received mFOLFOX6 (with or without bevacizumab), while FOLFIRI (with or without bevacizumab) was administered in nine patients (18%). Only three patients were treated with FOLFOXIRI plus bevacizumab (6%), whereas nine patients (18%) received other regimens (such as a fluoropyrimidine, alone or in combination with bevacizumab). Twenty patients (40%) received only one line of therapy, while ten patients (20%) received two lines, thirteen patients (26%) received three lines, five patients (10%) received four lines and, only two patients (4%) received five lines of therapy.

**Figure 1:**
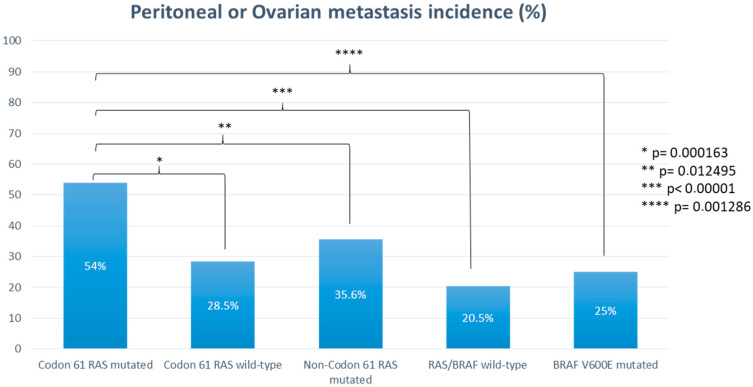
Probability of experiencing peritoneal or ovarian metastases according to *RAS* and *BRAF* mutational status.

The comparator dataset included 648 consecutive patients with codon 61 *RAS* wild-type mCRC treated at our institution during the same time frame. This group included 326 patients (50.3%) with an *RAS*-mutated disease not involving codon 61 (non-codon 61 *RAS*-mutated mCRCs), 254 patients (39.2%) with an *RAS* and *BRAF* wild-type disease (*RAS*/*BRAF* wild-type mCRCs), and 68 patients (10.5%) with a *BRAF* V600E-mutated disease (*BRAF* V600E-mutated mCRCs). The probability of experiencing peritoneal or ovarian metastases was statistically significantly higher in patients with codon 61 *RAS*-mutated mCRC than in patients with codon 61 *RAS* wild-type mCRC (54% vs. 28.5%, *p* = 0.000163) (Figure 1). More specifically, the rate of peritoneal or ovarian metastases was higher in the codon 61 *RAS*-mutated cohort also when compared to the non-codon 61 *RAS*-mutated cohort (54% vs. 35.6%, *p* = 0.012495), *BRAF* V600E-mutated cohort (54% vs. 25%, *p* = 0.001286), and *RAS*/*BRAF* all wild-type cohort (54% vs. 20.5%, *p* < 0.00001) (Figure 1).

**Figure 2:**
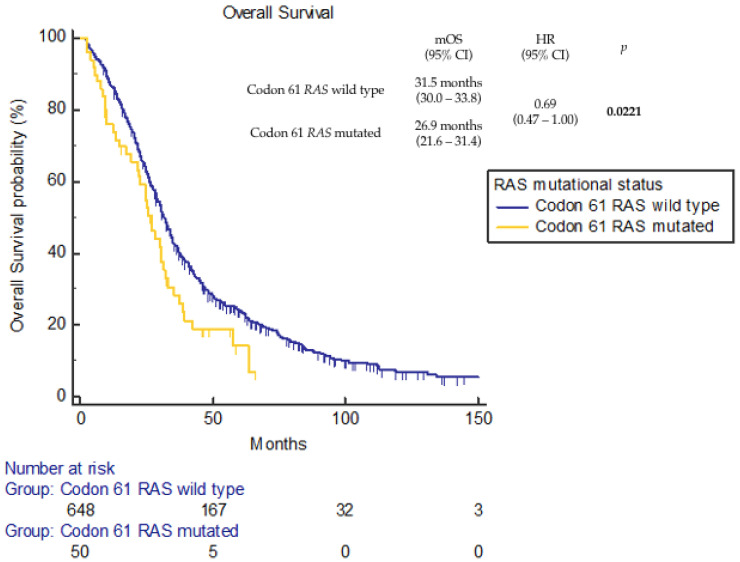
Overall survival according to codon 61 *RAS* mutational status.

At a median follow up of 96.2 months (95% confidence interval (CI), 92.4–109.0 months), 40 death events were reported in the codon 61 *RAS*-mutated cohort and 556 in the comparator dataset. Median OS (mOS) was 26.9 months (95%CI 21.6–31.4 months) for the codon 61 *RAS*-mutated cohort and 31.5 months (95%CI 30.0–33.8 months) for the codon 61 *RAS* wild-type dataset (hazard ratio (HR) 0.69, 95%CI 0.47–1.00; *p* = 0.0221) (Figure 2).

**Figure 3:**
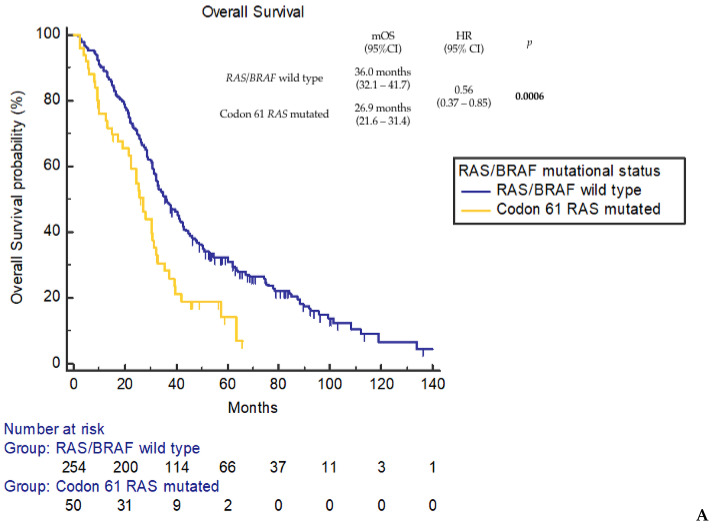

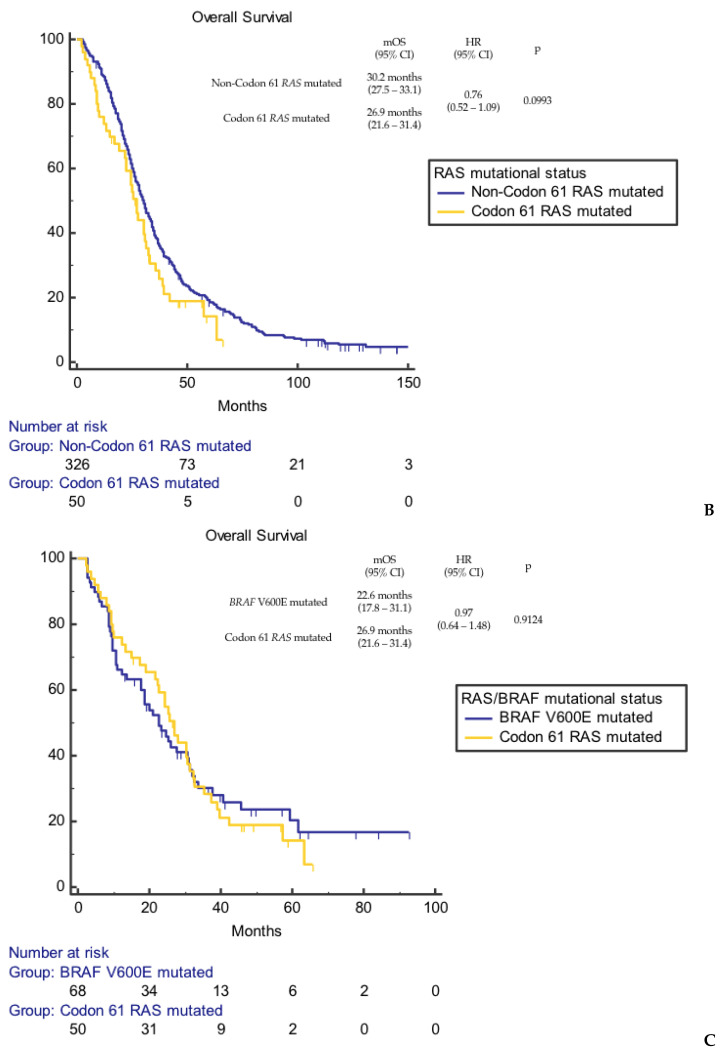
Overall survival according to codon 61 *RAS* mutational status. (**A**) Codon 61 *RAS*-mutated vs. *RAS*/*BRAF* wild-type patients. (**B**) Codon 61 *RAS*-mutated vs. non-codon 61 *RAS*-mutated patients. (**C**) Codon 61 *RAS*-mutated vs. *BRAF* V600E-mutated patients.

Moreover, dissecting the comparator dataset in accordance with *RAS* and *BRAF* mutational status, mOS was confirmed to be significantly shorter for the codon 61 *RAS*-mutated cohort compared to the *RAS* and *BRAF* wild-type cohort (mOS 36.0 months, 95%CI 32.1–41.7 months; HR 0.56, 95%CI 0.37–0.85; *p* = 0.0006) (Figure 3A). On the contrary, no statistically significant difference was observed compared to the non-codon 61 *RAS*-mutated cohort (mOS 30.2 months, 95%CI 27.5–33.1 months; HR 0.76, 95%CI 0.52–1.09; *p* = 0.0993) (Figure 3B) and the *BRAF* V600E-mutated cohort (mOS 22.6 months, 95%CI 17.8–31.1 months; HR 0.97, 95%CI 0.64–1.48; *p* = 0.9124) (Figure 3C).

## 4. Discussion

In our study, we demonstrated that codon 61 *RAS*-mutated mCRCs display a tropism for metastatic spread to the peritoneum and ovary and have a negative prognostic impact.

We found out that patients with mCRC harboring codon 61 *RAS* mutation are more likely to experience peritoneal or ovarian metastases during their clinical history. Indeed, the incidence of peritoneal or ovarian involvement was significantly higher in the codon 61 *RAS*-mutated cohort than in the comparator dataset including mCRC without codon 61 *RAS* mutations (54 vs. 28.5%, *p* = 0.000163). The higher tropism for the peritoneum and ovary of codon 61 *RAS*-mutated mCRCs retained statistical significance when compared to all molecular subgroups of the control dataset (*p* = 0.012495, *p* = 0.001286, and *p* < 0.00001, compared to non-codon 61 *RAS*-mutated, *BRAF* V600E-mutated, and *RAS*/*BRAF* all wild-type cohort, respectively). This feature might be related to the worse prognostic impact. To our knowledge, this is the first evidence of an impact of *RAS* mutational status on metastatization pattern in colorectal tumors. Although involving a small population, this evidence might lead to a more accurate surveillance for peritoneal spread, such as diagnostic laparoscopy before primary tumor resection or routine peritoneal washing sampling. Moreover, this evidence might have pivotal implication in the era of neoadjuvant treatment of colon cancer that we are currently approaching [27]. Confirmation of a peritoneal or ovarian tropism could support therapeutic approaches such as prophylactic hyperthermic intraperitoneal chemotherapy (HIPEC) in combination for stage II–III primary tumor resection or in combination with cytoreductive surgery (CRS) for a stage IV disease in this category of patients. Thus, whether this evidence were validated, codon 61 *RAS* status should be taken into account in a routine clinical approach and might be used as a stratification factor when planning surgical trials (either prophylactic or therapeutic). The COLOPEC trial failed to show the efficacy of adjuvant HIPEC with oxaliplatin, delivered at the time of primary tumor resection or within 5–8 weeks, for T4 or perforated stage II–III colon cancer [28]. Compared to the control arm, there was no difference in the peritoneal-free survival rate at 18-months [28]. Accordingly, the PROPHYLOCHIP trial did not show benefit in terms of disease free-survival for second surgical look combined with HIPEC compared to surveillance in patients at a high risk of developing peritoneal metastases [29]. Concerning stage IV disease, the PRODIGE 7 trial failed to show an additional benefit, in terms of OS and disease-free survival, of combining oxaliplatin-based HIPEC with CRS [30]. Based on this evidence, HIPEC is not currently recommended, neither in adjuvant settings nor in combination with CRS for stage IV disease [2,31]. We postulate that codon 61 *RAS* mutations might be used as stratification factors or even inclusion criteria to optimize the selection of patients that can benefit from adjuvant or therapeutic HIPEC in future studies.

Furthermore, recently published analyses of colorectal peritoneal metastases microenvironment demonstrated a predominance of the consensus molecular subtype (CMS) 4, which is associated with the infiltration of regulatory T cells and macrophages that inhibit immune response [32]. This could unveil a role for immunotherapy regimens in this setting in order to overcome these inhibitory mechanisms and to control peritoneal disease. Patterns of tumor-infiltrating lymphocyte expression in peritoneal nodes seem also to be associated with a better surgical outcome and improved OS, particularly in the case of low-volume disease, providing a possible patient selection for peritoneal cytoreductive surgery and HIPEC, as well as novel pathways for effective immunotherapy [33].

Our data showed a negative prognostic impact of codon 61 *RAS* mutations compared to *RAS*/*BRAF* wild-type disease, while no difference in terms of OS was observed compared to other non-codon 61 *RAS*-mutated tumors and *BRAF* V600E-mutated tumors. After a median follow up of 96.2 months (95%CI 92.4–109.0), median OS was significantly shorter in tumors harboring *RAS* codon 61 mutations compared to those harboring wild-type codon 61 (26.9 vs. 31.5 months, *p* = 0.0221). The negative prognostic impact of codon 61 *RAS* mutations was retained compared to *RAS*/*BRAF* wild-type tumors (26.9 vs. 36.0 months, *p* = 0.0006). This negative prognostic role in colon cancer differs from what is observed in other diseases such as pancreatic adenocarcinoma, where *RAS* codon 61 mutations showed a significantly improved survival [34].

We showed that mCRCs harboring *RAS* codon 61 mutations have distinct clinical and biological behaviors. This is of great interest given the high frequency of codon 61 *RAS* mutations as mechanism of secondary resistance to anti-EGFR agents and the advent of RAS inhibitors [35]. The acquired *RAS* codon 61 mutations could play a role in developing resistance to EGFR inhibitors, being enriched in the setting of secondary resistance in mCRCs treated with anti-EGFR agents [36]. Notably, the incidence of acquired *RAS* codon 61 mutations differs according to the treatment line and to the administration in combination with doublet cytotoxic chemotherapy. Indeed, the analysis of paired plasma samples from patients with *RAS*/*BRAF* wild-type mCRC treated with anti-EGFR agents showed a low incidence of acquired *KRAS* codon 61 mutations in patients treated in the first line in combination with chemotherapy. On the contrary, patients treated with single-agent anti-EGFR in the third line were more likely to develop acquired mutations. Of those, 63% were *KRAS* codon 61 mutations [37]. In the CRICKET trial [38], *RAS* mutations were identified in 48% of liquid biopsy samples collected at the baseline of the anti-EGFR rechallenge; of those, 17% involved codon 61. Furthermore, codon 61 variants have been recently identified with high frequency in the ctDNA of patients with mCRC with secondary resistance to anti-EGFR agents [18,19,20,21], with a prevalence of 50% in the CHRONOS trial [18].

Despite many years of effort, only lately have anti-RAS therapies reached clinical application. This is probably linked to the great complexity of RAS, not only in CRC but also in other tumors. The *RAS* gene isoforms display notable variations in the frequency of mutations at each of the three hotspots (G12, G13, and Q61), which have distinct structural and biochemical defects [39]. Recently, novel KRAS G12C inhibitors, alone or in combination with EGFR inhibitors, showed promising results [40,41,42,43,44]. Finally, the phase III CodeBreaK 300 trial showed that dual KRAS G12C and EGFR blockade with sotorasib and panitumumab in refractory *RAS* G12C-mutated mCRCs is associated with longer progression-free survival and a higher response rate than the standard treatment [45].

Our study has several limitations. First of all, the small sample size and the mono-institutional design do not allow us to extend our conclusions to the general population. Nevertheless, we should point out that this is the widest codon 61 *RAS*-mutated cohort reported so far. Moreover, selection biases are inevitable, given the retrospective nature of our analysis. Wider, multicentric, and prospective trials are warranted to confirm our results and investigate the possible prophylactic and therapeutic implications.

## 5. Conclusions

In our study, we identified a statistically significant correlation between codon 61 *RAS* mutations and metastatic involvement of the peritoneum and ovary. This is the first evidence of an impact of *RAS* mutational status on the metastatization pattern in colorectal tumors. This evidence could lead to new prophylactic applications in preventing peritoneal spreading in this specific group of patients.

Differently to what we have thought for years, “not all RAS mutants are created equal”, as Hobbs et al. stated [36], and our aim in the future is to better characterize each of them, leading to new therapeutic strategies.

## Figures and Tables

**Table 1:** Codon 61 *RAS*-mutated patients characteristics.

Characteristics		N = 50 (%)	*KRAS* (n = 28)	*NRAS* (n = 22)
**Age (at metastatic diagnosis),** **median (range)**		65 yrs (34–86 yrs)	65 yrs (41–86 yrs)	63 yrs (34–84 yrs)
**ECOG PS**	**0**	25 (50%)	15 (53%)	10 (45%)
	**1**	19 (38%)	8 (28%)	11 (50%)
	**2**	6 (12%)	5 (19%)	1 (5%)
**Sex**	**Male**	19 (38%)	10 (36%)	9 (41%)
	**Female**	31 (62%)	18 (64%)	13 (59%)
**Previous surgery**	**Y**	40 (80%)	22 (79%)	18 (82%)
	**N**	10 (20%)	6 (21%)	4 (18%)
**Metastatic at diagnosis**	**Y**	33 (66%)	19 (68%)	14 (64%)
	**N**	17 (34%)	9 (32%)	8 (36%)
**Primary tumor location**	**Right**	14 (28%)	8 (29%)	6 (27%)
	**Left**	36 (72%)	20 (71%)	16 (73%)
**Sites of metastatic disease at diagnosis**	**Liver**	24 (48%)	12 (43%)	12 (54%)
	**Lung**	11 (22%)	7 (25%)	4 (18%)
	**Nodes**	15 (30%)	8 (28%)	7 (32%)
	**Peritoneum/Ovary**	16 (32%)	7 (25%)	9 (41%)
	**Other**	5 (10%)	3 (10%)	2 (9%)
**Peritoneal and/or ovarian metastasis**	**Y**	27 (54%)	13 (46%)	14 (64%)
	**N**	23 (46%)	15 (54%)	8 (36%)
**First line chemotherapy regimen**	**FOLFOXIRI +/− bevacizumab**	3 (6%)	0	3 (14%)
	**FOLFOX +/− bevacizumab**	29 (58%)	20 (71%)	9 (41%)
	**FOLFIRI +/− bevacizumab**	9 (18%)	3 (11%)	6 (27%)
	**Other**	9 (18%)	5 (18%)	4 (18%)
**Total number of treatment lines**	**1**	20 (40%)	15 (53%)	5 (23%)
	**2**	10 (20%)	5 (18%)	5 (23%)
	**3**	13 (26%)	6 (21%)	7 (32%)
	**4**	5 (10%)	1 (4%)	4 (18%)
	**5**	2 (4%)	1 (4%)	1 (4%)
***RAS* mutation**	***KRAS***	28 (56%)		
	Q61X	15 (30%)		
	Q61H	6 (12%)		
	Q61L	3 (6%)		
	Q61R	2 (4%)		
	G61X	2 (4%)		
	***NRAS***	22 (44%)		
	Q61R	8 (16%)		
	Q61K	8 (16%)		
	Q61L	5 (10%)		
	G61H	1 (2%)		

ECOG PS: Eastern Cooperative Oncology Group performance status; N: no; Y: yes; yrs: years.

## Data Availability

Data are contained within the article.
